# Transcriptome Sequencing and Analysis of the Fast Growing Shoots of Moso Bamboo (*Phyllostachys edulis*)

**DOI:** 10.1371/journal.pone.0078944

**Published:** 2013-11-07

**Authors:** Zhenhua Peng, Chunling Zhang, Ying Zhang, Tao Hu, Shaohua Mu, Xueping Li, Jian Gao

**Affiliations:** International Center for Bamboo and Rattan, Key Laboratory of Bamboo and Rattan Science and Technology, State Forestry Administration, Beijing, People's Republic of China; Nanjing Forestry University, China

## Abstract

**Background:**

The moso bamboo, a large woody bamboo with the highest ecological, economic, and cultural value of all bamboos, has one of the highest growth speeds in the world. Genetic research into moso bamboo has been scarce, partly because of the lack of previous genomic resources. In the present study, for the first time, we performed *de novo* transcriptome sequencing and mapped to the moso bamboo genomic resources (reference genome and genes) to produce a comprehensive dataset for the fast growing shoots of moso bamboo.

**Results:**

The fast growing shoots mixed with six different heights and culms after leaf expansion of moso bamboo transcriptome were sequenced using the Illumina HiSeq™ 2000 sequencing platform, respectively. More than 80 million reads including 65,045,670 and 68,431,884 clean reads were produced in the two libraries. More than 81% of the reads were matched to the reference genome, and nearly 50% of the reads were matched to the reference genes. The genes with log 2 ratio > 2 or < −2 (P<0.001) were characterized as the most differentially expressed genes. 6,076 up-regulated and 4,613 down-regulated genes were classified into functional categories. Candidate genes which mainly involved transcript factors, plant hormones, cell cycle regulation, cell wall metabolism and cell morphogenesis genes were further analyzed and they may form a network that regulates the fast growth of moso bamboo shoots.

**Conclusion:**

Firstly, our data provides the most comprehensive transcriptomic resource for moso bamboo to date. Candidate genes have been identified and they are potentially involved in the growth and development of moso bamboo. The results give a better insight into the mechanisms of moso bamboo shoots rapid growth and provide gene resources for improving plant growth.

## Introduction

Bamboo is one of the most important non-timber forest plants in the world. About 2.5 billion people depend on it economically, with a trade value of more than 2.5 billion US dollars per year [1], [2]. It is also one of the most important forest resources because of its rapid growth rate, unique strength, and its capacity to easily adapt. On average, a large number of bamboo species reach their maximum height of 15–30 m in 2–4 months and reach full maturity in about 3–8 years [3].

Moso bamboo, *Phyllostachys edulis* (Carrière) J. Houzeau (Synonym *Phyllostachys heterocycla* Carrière) [4], is a large woody bamboo with the highest ecological, economic, and cultural value of all bamboos in Asia, accounting for up to 70% of the total area of bamboo growth. It has been valued at 5 billion US dollars of annual forest production in China. Its striking growth speed makes it one of the fastest growing plants in the world. The growth of its shoot is rapid and steady and in suitable spring conditions, at the peak of its growth, the shoot can grow as long as 100 cm within 24 hours, and reach its maximum height of about 20 meters in 45 to 60 days [2], [5].

To explore the mystery of the rapid growth of bamboo which has attracted researchers' interest, mounting studies have focused on the general mode of growth, anatomical structure of the culms [6]–[8], and sequential elongation of the internodes from the base to the top [9]. Several putative related genes involved in shoot growth such as *SuS*, *PAL* and *SUT* have been identified from certain bamboo species [10]–[14]. Recently, many genomic studies in bamboo have been conducted including the sequencing of a set of cDNAs [5], [10], [15]–[17], ESTs [18], [19], generation of a monoclonal antibody bank [20], chloroplast genome sequencing [21], identification of synthetic genes between bamboo and other grasses [22], phylogenetic analysis of *Bambusoideae* subspecies [23], genetic diversity analysis of 23 bamboo species [24], and the identification of different bamboo species [25]. A proteomics study showed that many metabolic processes of cell wall structure were employed in the fast growth of bamboo culms [9]. One transcriptome of several tissues (seeds, flowers and tissues including leaves, stem, shoots and root) in *Dendrocalamus latiflorus* was analyzed and genes encoding eight key enzymes, plant hormones, and involved in lignin biosynthesis, growth and development were identified [26]. Despite vigorous previous efforts to identify genetic factors in the growth and development, a comprehensive description of moso bamboo transcriptome remains unavailable, and the molecular mechanism underlying its rapid growth has not been fully elucidated. With the announcement of the genome sequence of moso bamboo [2], it is feasible and reliable to identify and determine the molecular regulation mechanisms of all functional genes in the *P*. *edulis*.

Transcriptome sequencing is a convenient way to rapidly obtain information on the expressed fraction of genome, which provides information on gene expression, gene regulation, and amino acid content of proteins. Therefore transcriptome analysis is essential for interpreting the functional elements of the genome and revealing the molecular constituents of cells and tissues [27]–[29]. In the present study, we performed *de novo* transcriptome sequencing for moso bamboo with the Illumina HiSeq™ 2000 sequencing platform. A total of 10, 689 differentially expressed genes were identified in the sequencing pool. For the first time, we have comprehensively characterized the molecular basis of the physiological processes during the fast growth of moso bamboo shoots and provided potential gene candidates for further research. We believe that this new dataset and gene screening list will be a useful resource for future genetic and genomic studies on this species.

## Results

### Sequence analysis and assembly

To obtain a global view of the moso bamboo transeriptome and identify genes involved in shoot initiation, a cDNA library from bamboo culms after leaf expansion (abbreviated to CK) and a mixed cDNA library from 6 heights (abbreviated to H) were constructed and sequenced using the Illumina HiSeq™ 2000 sequencing platform. More than 65 million total reads from each of the CK and H libraries were acquired including 65,045,670 and 68,431,884 clean reads, and the total Base Pairs produced were 5,854,110,300 and 6,158,869,560 in each library respectively (Table 1).

**Table 1:** Alignment statistics results of library CK and H.

Sample	CK		H	
Total reads	65045670		68431884	
Total base pairs	6147462240		6158869560	
**Map to genome**	**Reads number**	**Percentage**	**Reads number**	**Percentage**
Total mapped reads	52805612	81.18%	55520086	81.13%
Perfect match	41749711	64.19%	44359624	64.82%
< = 5 bp mismatch	11055901	17.00%	11160462	16.31%
Unique match	47847557	73.32%	51402531	75.11%
Multi-position match	5114710	7.86%	4117555	6.02%
Total unmapped reads	12240058	18.82%	12911798	18.87%
**Map to Gene**				
Total mapped reads	30557906	46.98%	33371577	48.77%
Perfect match	22766209	35.00%	25062364	36.62%
< = 5 bp mismatch	7791697	11.98%	8309213	12.14%
Unique match	29496519	45.35%	32276895	47.17%
Multi-position match	1061387	1.63%	1094682	1.60%
Total unmapped reads	34487764	53.02%	35060307	51.23%

### The reads mapping to the reference genome dataset

To identify the genes corresponding to these clean reads in each library, the clean reads were mapped to the reference genes expressed in the moso bamboo genome. Mapping results showed that 64.19% (41,749,711) and 64.82% (44,359,624) reads from each library were perfectly matched to the reference genome while about 35.00% (22,766,209) and 36.62% (25,062,364) were perfectly matched to the reference genes (Table 1). The percentages of unique matched to genome were 73.32% (47,847,557) and 75.11% (51,402,531), and those matched to reference genes were 45.35% (29,496,519) and 47.17% (32,276,895) in the two libraries. Altogether, there were 52,805,612 (81.18%) and 55,520,086 (81.13%) reads matched to the reference genome, and 30,557,906 (46.98%) and 33,371,577 (48.77%) reads matched to the reference genes. However, as a result of the significant sequencing depth of Illumina technology and incomplete annotation of the moso bamboo genome, 18.82% and 18.87% unmatched reads to the reference genome and 53.02% and 51.23% unmatched reads to the reference genes in each library were observed. The alignment statistics results of these reads from library CK and H are also shown in Table 1.

**Figure 1:**
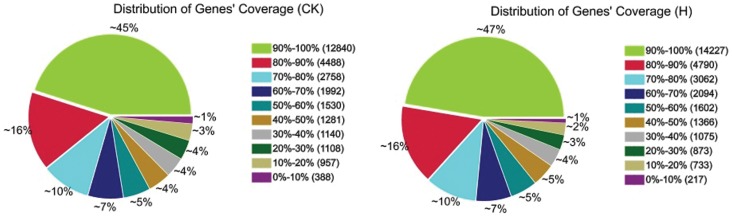
Distribution of gene's coverage identification for differentially expressed genes.

Gene coverage was calculated as the percentage of a gene covered by reads from each of the CK and H libraries, with 12,840 and 14,427 genes showing 45% and 47% of total genes with coverage between 90%–100%, 4,488 and 4,790 genes showing 16% of total genes with coverage between 80%–90% respectively (Figure 1). Randomness of reads mapped to reference genome and genes are shown in Figure S1.

**Figure 2:**
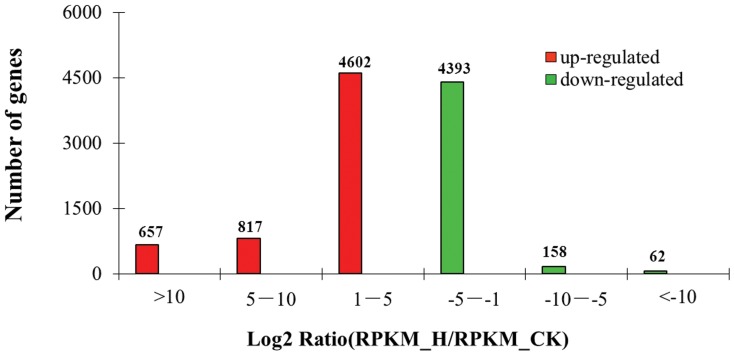
The distribution of fold-changes in differentially expressed gene numbers.

By comparing the two libraries at a statistically significant value (P<0.01), 30,515 genes were detected changing expression. The majority of genes were expressed at similar levels in the two libraries: approximately 85.35% genes showed a<5- fold difference in expression, yet a great number of differentially expressed genes (DEGs) were identified. 3754 genes with expressional changes in the range 5–10-fold accounted for 12.30%, and only 2.35% (719–657 up-regulated, 62 down-regulated) genes showed > 10-fold changes in expression level. 10,689 most differentially expressed genes represented 6,076 up-regulated and 4,613 down-regulated genes. It is of note that, among 719 genes showed > 10-fold changes in expression level, 657 genes were up-regulated, while only 62 genes showed to be down-regulated. The distribution of fold-changes in gene number between the two libraries is shown in Figure 2. For the study, the 10,689 most differentially expressed genes were analyzed further and the key gene sources of molecular basis that underline the fast growth traits of shoots that particularly interested us were identified according to their expression levels and gene numbers.

### Gene ontology (GO) annotation and KEGG pathway category of DEGs

A total of 16,519 genes were categorized into the three main categories of the GO classification with 37.1% (6,123) for cellular components, 31.5% (5,201) for molecular functions, and 31.4% (5,195) for biological processes. For cellular components, genes involved in cell (GO: 0005623, 5774, 94.9%) and cell part (GO: 0044464, 5774, 94.9%) were most highly represented, followed by intracellular components with 5,390 genes (GO: 0005622, 88.0%). For molecular functions, catalytic activity (GO: 0003824, 3513, 67.5%) were the most representative of GO term, followed by binding (GO: 0005488, 3229, 62.1%) and organic cyclic compound binding (GO: 0097159). Heterocyclic compound binding (GO: 1901363) shared the same percentages of 39.2% representing 2041 genes. Regarding biological processes, the most represented category was localization (GO: 0051179, 998, 19.2%), followed by cellular aromatic compound metabolic process (GO: 0006725) with 993 genes and 19.1%. Establishment of localization (GO: 0051234, 962, 18.5%) was the third most highly represented category (Table S1).

A total of 8,342 genes mapped into 126 pathways were picked after this process. A summary of the genes involved in these pathways has been included in Table S2. The largest category was metabolic pathways (Ko01100, 29.31%) with which 2445 genes were annotated, followed by RNA transport (Ko03013, 1682 genes, 20.16%), mRNA surveillance pathway (Ko03015, 1464 genes, 17.55%), biosynthesis of secondary metabolites (Ko01110, 911 genes, 10.92%), glycerophospholipid metabolism (Ko00564, 812 genes, 9.73%), endocytosis (Ko04144, 794 genes, 9.52%), ether lipid metabolism (Ko00565, 761, 9.12%), plant-pathogen interaction (Ko04626, 716 genes, 8.58%) and plant hormone signal transduction (Ko04075, 593 genes, 7.5%)(Table S2).

### Quantitative real-time PCR (qRT-PCR) evaluation

As shown in Table 2, some transcripts from highly abundant Illumina genes appeared at the expected lower numbers in the qRT-PCR analyses. For example, *SAUR* showed a 14-fold increase in the Illumina analysis, whereas it was significantly up-regulated by 109-fold in the RT-PCR analysis. However, as expected, the general expression patterns of transcripts from the Illumina sequencing are basically close to the results from qRT-PCR, also confirming the reliability of our transcriptome analysis.

**Table 2:** Evaluation of the expression profiles variation for randomly selected genes between Illumina sequencing by qRT-PCR.

Gene	Gene ID	Description	Illumina H/CK^a^	qRT-PCR H/CK^b^
*CYCA*	PH01002854G0180	cyclin A	6	10
*EXP*	PH01002238G0310	expansin	13	3
*FTK*	PH01001577G0100	fructokinase	20	53
*BGL*	PH01001888G0390	Beta glucanase like	9	2
*ARF*	PH01000057G1420	auxin response factor	3	5
*MYB*	PH01002707G0220	myb proto-oncogene protein	10	27
*MYC*	PH01000201G0400	transcription factor MYC	13	2
*Dof*	PH01000664G0640	dof zinc finger protein 10	6	10
*SAUR*	PH01004171G0020	mall auxin up RNA	14	109
*AUX1*	PH01000373G0290	auxin influx carrier (AUX1 LAX family)	12	49
*GID*	PH01000436G0270	gibberellin receptor GID	−12	0.01
*GID1*	PH01000068G0110	gibberellin receptor GID1	15	2

a Ratio of log2 (RPKM_H/RPKM_CK).

b Ratio of relative concentrations.

**Figure 3:**
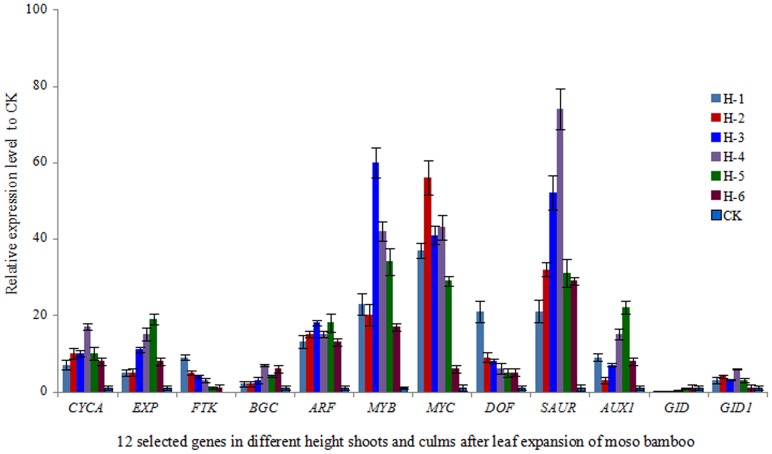
The expression profiles of 12 selected genes in different height shoots and culms after leaf expansion of moso bamboo. The transcript levels were normalized to that of *TIP41* (tonoplast intrinsic protein 41), and the level of each gene in the control was set at 1.0. Error bars represent the SD for three independent experiments. H-1, shoots of 10 cm, H-2, shoots of 50 cm, H-3, shoots of 100 cm, H-4, shoots of 300 cm, H-5, shoots of 600 cm, H-6, shoots of 900 cm, CK, culms those have stopped growing in height after leaf expansion. *CYCA* (cyclin A), *EXP* (expansin), *FTK* (fructokinase), *BGL* (beta-glucanase like), *ARF* (auxin response factor), *MYB* (myb proto-oncogene protein), *MYC* (transcription factor MYC), *DOF* (dof zinc finger protein 10), *SAUR* (small auxin up RNA), *AUX1* (auxin influx carrier LAX family), *GID* (gibberellin receptor GID), *GID1* (gibberellin receptor GID1).

According to the qRT-PCR results (Figure 3), *FTK* (fructokinase) and *DOF* (dof zinc finger protein 10) genes were down-regulated, whereas *GID* (gibberellin receptor GID) gene up-regulated as the shoot grew. Other genes such as *CYCA* (cyclin A), *EXP* (expansin), *MYB* (myb proto-oncogene protein), *MYC* (transcription factor MYC), and *SAUR* (small auxin up RNA), were first induced to a high expression level and then decreased, while the expression of *BGL* (beta-glucanase like), *ARF* (auxin response factor), *AUX1* (auxin influx carrier LAX family), *GID*1 (gibberellin receptor GID1) fluctuated during the growth of moso bamboo shoots. Generally, the results obtained also agreed with the Illumina analysis.

### Putative transcription factors involved in growth

We identified a total of 392 genes according to their high expression levels through BLASTn analysis, which implied that those genes may have putative functions as transcription or relative factors including those belonging to or related to the E2F family, MYB family, MYC family, WRKY family, TGA family, F-box family, MADSbox family *et al* (Table 3, Table S4). As the table listed, the MYB family, the MYC family, the WRKY family, the F-box family and the zinc finger protein family had the largest numbers of up-regulated genes. Five putative genes encoding the E2F family were all up-regulated. The F-box family had more up-regulated than down-regulated genes. The MYC family had 45 putative genes and many of them had a high expression level. The WRKY family and the TGA family presented more down-regulated genes than up-regulated. The ARF family, whose expression levels were all lower than 3-fold, also had more down-regulated than up-regulated genes.

**Table 3:** Putative genes involved in fast growth of moso bamboo shoots.

Putative genes	Total gene numbers	Up-regulated gene numbers	Down-regulated gene numbers
***Transcription factor families***			
Auxin response factor (ARF)	22	6	16
E2F transcription factor (E2F)	5	5	0
MYB transcription factor (MYB)	98	67	31
Transcription factor MYC (MYC)	45	30	15
WRKY transcription factor (WRKY)	54	16	38
Growth-regulating factor (GDF)	2	2	0
Transcription factor TGA (TGA)	23	9	14
F-box	46	29	17
MADS-box transcription factor (MADS)	13	5	8
High mobility group box (HMG)	8	7	1
NAC transcription factor (NAC)	10	8	2
WD repeat-containing protein (WD)	6	2	4
Zinc finger protein	46	16	30
***Plant hormones***			
Auxin	169	103	66
Cytokinin	82	49	33
Gibberellin	57	30	27
Abscisic acid	210	88	122
Ethylene	88	47	41
Brassinosteroid	202	135	67
Salicylic acid	103	41	62
Jasmonate	32	21	9
Polyamine	12	8	4
Peptide	199	95	104
***Cell cycle regulation***			
Cyclin A(CYCA)	7	6	1
Cyclin B(CYCB)	7	7	0
Cyclin D(CYCD)	14	12	2
Cell division cycle(CDC)	19	14	5
Cyclin-dependent kinase(CDK)	17	12	5
Cyclin-dependent kinase inhibitor(CKI)	1	1	0
KIp-related protein(KRP)	1	1	0
Retinoblastoma-like(RB)	3	3	0
***Cell wall metabolism andcell morphogenesis***			
Expansin (EXP)	8	8	0
Xyloglucan Endotransglucosylase/hydrolase (XET/XTH)	6	6	0
Xyloglucan fucosyltransferase (XF)	1	1	0
UDP-glycosyltransferase (UGT)	18	11	7
Hydroxycinnamoyl-CoA: shikimate/quinAte hydroxycinnamoyltransferase (HCT)	23	17	6
Cellulose synthase A (CesA)	10	9	1
Cellulose synthase-like(CSL)	25	23	2
4-coumarate-CoA ligase (4CL)	11	3	8
Cinnamoyl-CoA reductase (CCR)	19	12	7
Phenylalanine ammonia-lyase (PAL)	7	6	1
Beta-glucosidase-like(BGL)	45	37	8
Endoglucanase	23	23	0
Beta glucanase	3	3	0
Beta-1,3-glucanase	3	2	1
Laccase	6	6	0
Polygalacturonase	23	22	1
Pectate lyase	4	4	0
Chitinase	13	12	1
Sucrose synthase	13	5	8
Sugar transporter	28	24	5
Cortical cell-delineating protein(CCD)	2	2	0

### Putative gene encoding or related to plant hormones

Altogether, there were 1,154 genes encoding 6 main plant hormones and other related plant hormones, identified in the dataset (Table 3, Table S4). Among these genes which accounted for about 11% of all the DEGs, 210 genes, the largest number among all pathways, were for the abscisic acid (ABA) pathway, a sort of plant hormones that has a passive effect on plant growth; there were only 169 genes involved in the auxin pathway, 82 for the cytokinin (CTK) pathway and 57 for gibberellin (GA) pathway, sorts of plant hormones those have positive effects on plant growth. Moreover, the brassinosteroid (BRs) pathway (202 genes) was the second highest category, which was followed by 199 genes related to the peptide pathway; genes related to the salicylic acid pathway involved 103 genes; 88 genes were for the ethylene pathway. All of the above demonstrated that these plant hormones play vital roles in the growth of shoot making moso bamboo even though bamboos are particular with other plants; 32 genes related to the jasmonate pathway and 12 genes for the Polyamine pathway, also indicating that they are both important to the growth of shoots.

### Putative genes involved in cell cycle regulation

Plant cell cycle regulatory factors such as cyclin A, cyclin B, cyclin D *et al*, could be found in data collection (Table 3, Table S4). Among them, cyclin A, cyclin B and cyclin D mainly composed of cyclins had higher up-regulated expression levels. Among 19 genes found in cell division cycle, 14 were up-regulated and 5 were down-regulated. The rest of the factors listed in Table 3 all presented normal expression levels: below 5-fold values, such as cyclin-dependent kinase (CDK), cyclin-dependent kinase inhibitor (CKI), KIp-related protein (KRP) and retinoblastoma-like (RB).

### Putative genes involved in cell wall metabolism and cell morphogenesis

A great number of putative transcripts were contained in our dataset, and selected key genes were listed in Table 3 and Table S4. The tables suggested that, genes participating in cell wall degradation, cell wall biosynthesis and cell morphogenesis encoding expansin, xyloglucan endotransglucosylase/hydrolase, xyloglucan fucosyltransferase, endoglucanase, pectin lyase cellulose synthase, COBRA and chitinase genes were greatly up-regulated, an outcome different from the maize brace roots [30]. Meanwhile, genes involved in Cinnamoyl-CoA reductase, 1, 3-beta-glucosidase-like and sugar transporter were not obviously up-regulated.

It is well known that the lignin content of bamboo is higher than most herbaceous plants. That may be the reason for differences in the numbers or levels of expressions of key enzymes involved in lignin biosynthesis [26], [31]. According to Table 4, genes encoding HCT, CesA and CSL which are key enzymes involved in lignin biosynthesis [31]–[33] had higher numbers and levels of expression, while PAL and laccase remained at a low level. Meanwhile, we compared the numbers of the seven key enzymes involved in lignin biosynthesis with the ma bamboo transcriptome, and moso bamboo genes identified from the genome sequences (Table 4). Peng *et al* predicted 6 copies encoding 4CL and 3 copies encoding CCR from the moso bamboo genome database [2]. However, our results revealed that these two genes were the most abundant ones that correlated with the results of ma bamboo [26]. We also could not scan genes for A1dOMT as can be seen in Liu's results. Moreover, whilst they both exist in the ma and moso bamboo genome, CCoAOMT and C4H could not be detected in our collection.

**Table 4:** Number of genes found in the ma bamboo and moso bamboo transcriptome and moso bamboo genome that encode seven key enzymes in the lignin biosynthesis pathway.

Enzymes	Moso bamboo	Ma bamboo^a^	Moso bamboo b
4-coumarate-CoA ligase (4CL)	11	35	6
Caffeoyl caffeoyl-CoA O-methyltransferase (CCoAOMT)	0	4	2
Cinnamoyl-CoA reductase (CCR)	19	10	3
Caffeic acid O-methyltransferase (COMT)	1	7	1
Cinnamate-4-hydroxylase (C4H)	0	4	4
Cinnamoyl alcohol dehydrogenase (CAD)	4	2	1
5-hydroxyconiferyl aldehyde O-methyltransferase (AldOMT)	0	0	7

a The results were cited from Liu *et al*. (2012).

b The results were cited from Peng *et al*. (2013).

## Discussion

### Genome database mapping in Illumina HiSeq™ 2000 sequence

The major goal of the present study is to preliminarily filter the key genes involved in the fast growth of moso bamboo shoots, as well as to provide groundwork for investigating regulating mechanisms of relevant genes. Our study firstly reported the transcriptional changes during the fast growth of shoot using the Illumina HiSeq™ 2000 sequencing platform combined with the moso bamboo genome database as a reference. Transcriptome analysis is essential in interpreting the functional elements of the genome and reveals the molecular constituents of cells and tissues [27], [28]. Several previous studies of the plant development transcript profiles using deep sequencing have been reported [26], [30], [34]–[36]. The genome reference is important for mapping in the transcriptome database as it makes the data more precise and comprehensive and sharply reduces the number of genes [30], [37]. Analysis and identification become much easier and more precise with a genome reference. In our collection, 10,689 differentially expressed genes were identified via mapping to the moso bamboo genome reference, just the same species, much less than the 68,229 unigenes identified in ma bamboo transcriptome and 80,418 transcripts in the floral transcriptome of ma bamboo through aligning with other species [26], [38]. It makes the data more reliable and the identification of the putative genes simpler than before, so more time and effort can be spent on further meaningful research, rather than complex analysis for sequencing. Additionally, the annotation of the reads is a crucial step in deep sequencing studies. We generated a 21 bp short read for mapping to the genome database in the present study, more specific than the 14 or 19 bp in previous serial analyses of gene expression [39]–[41]. Altogether, over 81% genes could be mapped to unique or non-unique positions, similar with the proportion described by Wang *et al*. and Liu *et al*. [26], [35]. Plus, the expression profiles of 12 selected genes were assessed by quantitative real-time PCR, and the results obtained revealed good correlation with the Illumina analysis. All of the above demonstrates that Illumina sequencing and read mapping are feasible with great accuracy in large moso bamboo genome. However, it still suffers limitations in the reference gene databases, because the sequence is deep and many genes have not been identified and annotated in the moso bamboo genome. In the present study, only less than 82% of genes were annotated leaving 1,151 highly expressed genes unanalyzed, which may play great role in the fast growth of shoots, show a necessity for further investigation.

### Transcription factors correlating with cell cycle and plant hormones

It has been reported that many transcription factor families play vital roles in plant growth, development and immunity [42]–[46]. Genes belonging to the zinc finger protein family, the WD repeat-containing protein family and the MADSbox family thought to correlate with bamboo flowering were also identified in the present study similar to those of ma bamboo [26], [47]. The MYB family, the WRKY family, the TGA family and the NAC family, on the other hand, are sigificant in that they respond to the plant's environment by responding to plant hormones, especially abscisic acid [48]–[51]. The genes encoding them were also deselected in our study. These results imply that these transcription factors may not only correlate with bamboo flowering or response to environment, but also play specific and diverse roles in regulating gene expression levels endowing bamboo with a unique rapid growth rate which acquires further studies by responding to plant hormones. In addition, the changes of the number and expression level of genes encoding these transcription factors and those genes encoding plant hormones confirmed our assumption. The E2F transcription factors correlating with cell cycle regulation play decisive roles in cell size [52], [53]. Their high expression level correlates to the regulatory factors of the cell cycle. It indicates that the E2F transcription factors may participate in the growth of shoots though co-regulation with the cell cycle regulatory factors. As low abundance expressed genes, the ARF family (Auxin response factors) plays various roles during the development of different plants, such as *Arabidopsis*, rice, poplar, maize, tomato [54]–[58]. *AtARF5* acts an important role in maintaining the apical meristem through directly regulating the expressions of cytokinin response gene *AtARR7* and *AtARR15* in *Arabidopsis* [59]. The expression changes of the ARF family suggest that these genes may also play different roles during the fast growth of shoots, which need further genome-wide analysis.

### Plant hormones in cell wall extensity

The previous studies have reported that the growth and development of plants depends on accretion, division and differentiation of cells, which are interrelated with plant cell cycle regulation [60]–[62]. Plant hormones, as is well known, determine the formation of flowers, stems, leaves, the shedding of leaves, the development and ripening of fruit [26], and they promote the cell wall expansion and growth by regulating the expression of cell wall expansion factors and reconstructing the cell wall polysaccharide network structure [63]. The auxin can quickly increase the scalability of the cell wall and the cell growth, by regulating expansions and wall-loosening protein expression though the auxin polar transport [64]–[67]; GA regulates the growth of the cell wall through the regulation of expression and living of cell wall structure modified enzymes [68]–[71]; BRs increases the extension of the cell wall by regulating the enzymes correlated with cell wall enzymes [72], [73]; many cell wall genes are regulated by ethylene [74]–[76]. Previous studies have reported that the elongation of rice stems is impacted by gibberellin, auxin, abscisic acid and ethylene, to which gibberellin is most closely impacted via mediating the expressions of *EUI1* gene, and the expansin genes which are the key factor in promoting cell wall expansion and growth and improving plant elongation [67], [77], [78]. A recent proteomics study showed that many metabolic processes of cell wall structure were employed in the fast growth of bamboo culms [2], [9]. Therefore, genes related to plant hormones and cell wall biosynthesis and cell morphogenesis were selected for the present study. In this study, the common ground between cell wall expansion factors and enzymes involved in the reports is the large quantity of genes numbers and high expression levels, especially expansin (EXP) and xyloglucan endotransglucosylase / hydrolase (XTH). Genes associated with plant hormones had the same trait. It implies that most of these genes may have reactions with each other resulting in the fast growth of shoots. Moreover, the identification of genes relating to plant hormones and their further research will provide a theoretical basis on bamboo tissue culture, callus culture which seriously hinders the genetic engineering of bamboo [79].

### Key enzymes involved in lignin synthesis

Hamberger *et al.* have reported the genome-wide analyses of phenylpropanoid-related genes in *Populus trichocarpa*, *Arabidopsis thaliana* and *Oryza sativa*, and found some differences amongst the different species and tissues [80]. There were significant differences between the transcriptome and genome found from the comparison of the number of genes found in ma and moso bamboo transcriptome and moso bamboo genome that encode seven key enzymes in the lignin biosynthesis pathway. It is not surprising because the moso bamboo genome were just sequenced with leaves which were not the most representative tissues for high lignin content [2], while ma bamboo transcriptome libraries were constructed from shoots, leaves, roots, seeds and flowers, and moso bamboo transcriptome sequenced shoots including six heights at different developing periods [26]. Therefore, the spatial distributions of genes related to lignin biosynthesis also influence the results. In the study, the results may be the temporal contributions of gene related to lignin biosynthesis as they were selected from shoots by comparing with culms after leaf expansion in the transcriptome. The reason may be that some other genes encode alternative methyltransferases, instead of A1dOMT activity that needs further characterization. All of the results above indicate that lignin biosynthesis in moso bamboo may follow unknown routes or pathways which cause lignin synthesis in moso bamboo displaying unique features. It will contribute to the wood formation of bamboo, subsequently providing guidance on improvement of wood properties of woody bamboo.

Moreover, genes encoding aquaporin PIP could be identified in our dataset. It has been reported that aquaporin PIP genes take part in the absorption of water and cell elongation, which is modulated by auxin [81], [82], and is a participant in cell growth that is positively regulated by gibberellin [83]. The finding of aquaporin PIP genes indicates that aquaporin PIP genes may play an important role in the fast growth of bamboo shoot via co-regulated by auxin and gibberellin.

### The significance of the candidate genes for bamboo breeding

In recent years, the deterioration of the global environment, greenhouse gas emissions and the energy crisis have created a bottleneck effect on economic development of countries all over the world. In China, forest resources are relatively lacking, timber is even scarcer leading to the implementation of natural forest protection projects. To exploit fast-growing and high yielding wood resources is an effective way to overcome these issues, and many previous studies have focused on it already. Research on the development and growth of poplar, over multiple biological processes of wood formation have revealed to some extent, molecular mechanism of forest development, and also provide numbers of genes affecting cell division and tissue differentiation, related to primary growth and secondary growth, and possible regulatory factors during the development of timber [84]–[86]. Previous studies have shown that genes involved in transcript factors, plant hormones, cell wall metabolism and cell morphogenesis evidently reveal major roles in cotton fiber elongation [86]–[89]. Bamboo, as one of the fastest-growing plants on Earth, plays an important role in solving the contradiction between supply and demand of timber, and its development and utilization has attracted a high level of attention from around the world [9], [79]. Almost all previous studies in bamboo [9]–[14], [26] imply that the development of culms is dominated by cell division, cell elongation and cell cycle. Plant endogenous hormones, such as cyclin A, cyclin B, auxin, and gibberellin *et al.*, appeared to strongly influence the cell division, cell elongation and cell cycle. In the present study, putative genes related to the genes mentioned above were also identified and analyzed, and the majority of them were highly differentially expressed. The results indicate that these putative genes may be closely related to the fast growth of moso bamboo shoots. Moreover, it has been confirmed by the altered expression profiles of 12 selected genes observed by qRT-PCR, which indicates that they were involved in the regulatory networks during the fast growth of moso bamboo shoots. For example, *FTK*, *DOF* and genes were up-regulated or down-regulated as the shoot grows, indicating that these genes play positive or negative roles in the speed of moso bamboo shoot growth. *CYCA*, *EXP*, *MYB*, *MYC*, *SAUR* genes' expression profiles indicate that they might play major roles in specific developmental stages. *BGL*, *ARF*, *AUX1*, *GID1* fluctuated during the growth of moso bamboo shoots, demonstrating that these genes might be regulated in a temporal manner. Generally speaking, all the above findings not only provide a theoretical basis for revealing the molecular regulation mechanism of the fast growth of moso bamboo shoots, understanding the structure of a single gene and carrying out the function of prediction and research, but also in practice particularly, offer candidate functional gene resources for improving bamboo growth even or other plants' fast growth through genetic engineering techniques, consequently provide more forest resources and improve the environment via the high-yielding forest tree cultivation.

## Conclusions

This study presents firstly the transcriptome sequencing analysis of mixed RNA from different heights of moso bamboo shoots via Illumina platform using the moso bamboo genome database as a reference. A large number of candidate genes involved in transcript factors, plant hormones, cell cycle regulation and cell wall metabolism and cell morphogenesis were identified which are worthy of further investigation. The limitation of our study is that the investigation only elucidates part of the picture and hence it is difficult to draw precise conclusions. However, the dataset will provide an important resource foundation for future genetic or genomes studies on bamboo species and will help to give better insight into the mechanism of the rapid growth of moso bamboo shoots and provide gene resources for improving bamboo growth even for the high-yielding forest tree cultivation.

## Materials and Methods

### Ethics Statement

All plant protocols were reviewed and approved by the International Center for Bamboo and Rattan and Key Laboratory of Bamboo and Rattan Science and Technology, State Forestry Administration. All necessary permits were obtained for the field studies from Anhui Provincial Academy of Forestry and Huoshan County Forestry Bureau, Lu'an City in Anhui Provence. The field work conducted for sampling did not affect the local ecology and did not involve endangered or protected species.

### Sample preparation and RNA extraction

**Figure 4:**
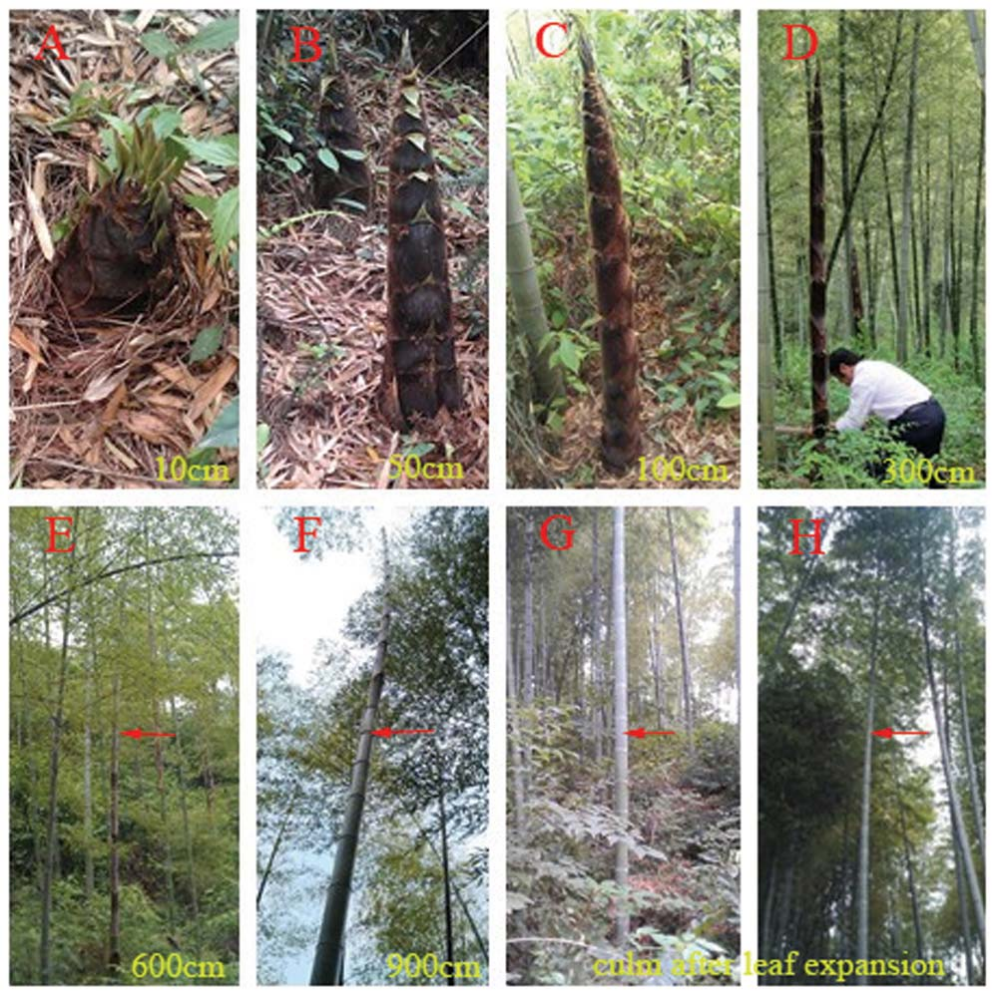
Different heights shoots and culms after leaf expansion of moso bamboo. A–F, H-1–H-6, different height shoots; G, H, CK, culms those have stopped growing in height after leaf expansion. The estimated shoot heights are given in the lower right corner of each panel. The positions indicated by red arrow were samples.

The moso bamboo samples were harvested in Huoshan County (E115°52′–116°32′; N31°03′–31°33′), Lu'an City in Anhui Provence in spring and autumn 2012. Six different height shoots (10, 50, 100, 300, 600, 900 cm) and culms after leaf expansion were selected according to the developmental stages of moso bamboo, labeled as H-1, H-2, H-3, H-4, H-5, H-6, and CK, respectively (Figure 4). Then, each shoot or culm was hewn and divided into basal, middle and top internodes based on its height by an equal division method. Subsequently, each sample was cut from the tissue located in the basal part of the three internodes above, and collected finally. The collected samples were immediately snap frozen in liquid nitrogen and stored at −80°C until further processing. Total RNA was isolated using the Trizol Reagent (Invitrogen). RNA quality was characterized initially on an agarose gel and *NanoDrop* 8000 spectrophotometer (*NanoDrop*, Thermo Scientific) and then further evaluated the integrity of RNA samples using Agilent 2100 Bioanalyzer, USA. All samples were collected absolute randomly and processed in sets of tri-plicates.

### cDNA library construction

The cDNA libraries of two different growth period samples (shoots mixed by 6 heights and culms after leaf expansion, each of which were combined with the tissues located in the basal part of the three internodes above) for transcriptome sequencing were constructed according to the manufacturer's instructions of Illumina's kit (Illumina, San Diego, CA). Briefly, magnetic beads with Oligo (dT) were used for isolating mRNA mixed with the fragmentation buffer, then the mRNA was fragmented into shorter fragments. Then cDNA was synthesized using the mRNA fragments as templates. Short fragments were purified and resolved with EB buffer for end reparation and single nucleotide A (adenine) addition. After that, the short fragments were connected with adapters. The suitable fragments were selected for the PCR amplification as templates. During the QC steps, the Agilent 2100 Bioanaylzer and the ABI StepOnePlus™ Real-Time PCR System were used in quantification and qualification of the sample library. Finally, the library was sequenced using Illumina HiSeq™ 2000 at Beijing Genomics Institute (BGI) in Shenzhen, China. All technical steps were performed in duplicate.

### Sequence data analysis and assembly

The original image data was transferred into sequence data as raw data or raw reads via base calling. Since the algorithms used in *de novo* transcriptome construction of the short reads provided by the Illumina platform may be severely inhibited by sequencing errors, a stringent cDNA sequence filtering process was employed to select clean reads. Firstly, reads with adaptors were removed. Second, reads with unknown nucleotides larger than 5% were removed. Secondly, low quality reads which the percentage of low quality bases (base quality≤20) was more than 20% were removed. Finally,the dataset was submitted and deposited in the repository of NIH Short Read Archive (the accession number is SRP029431). All the sequence data analysis and assembly was processed normally.

### Reads mapping to reference genome and genes

To identify the genes corresponding to the reads in each library, an essential dataset containing reference genes expressed in the moso bamboo genome was prepared. The moso bamboo reference genome and genes set were downloaded from the official website of the National Center of Genome Research (http://www.ncgr.ac.cn/bamboo) and NCBI site (http://www.ncbi.nlm.nih.gov/nuccore/FO203/436, http://www.ncbi.nlm.nih.gov/nuccore/FO203437, http://www.ncbi.nlm.nih.gov/nuccore/FO203443, http://www.ncbi.nlm.nih.gov/nuccore/FO203444, http://www.ncbi.nlm.nih.gov/nuccore/FO203447, http://www.ncbi.nlm.nih.gov/nuccore/FO203448, http://www.ncbi.nlm.nih.gov/nuccore/FO203439). After removing reads containing sequencing adapters and reads of low quality (reads containing Ns>5), clean reads were aligned to the moso bamboo genome using SOA Paligner/SOAP2 [90] allowing up to five mismatches.

### Identification of differentially expressed genes

To identify the genes associated with the growth of shoot and assess the molecular basis involved in the shoots' rapid growth, the expressional levels of genes were analyzed and the fold changes were assessed by the log2 ratio (RPKM-H/RPKM-CK). After the expressional abundances in each library were normalized to transcript per million (RPKM), then the most differentially regulated genes (differentially expressed genes, DEGs) with a log2 ratio (> 2 or <2) using a greater statistically significant value (P<0.001) as well as false discovery rates (FDR<0.01) were selected. Subsequently, based on sequence homology, these DEGs (differentially expressed genes) by gene ontology terms (http: //www. geneontology.org) were imported into Blast2GO [91], [92], a software package that retrieves GO terms, allowing gene functions to be determined and compared. These GO terms were assigned to query sequences, producing a broad overview of groups of genes catalogued in the transcriptome for each of three ontology vocabularies, biological processes, molecular functions and cellular components. Finally, to further demonstrate the usefulness of moso bamboo genes generated in the present study, we identified biochemical pathways represented by the gene collection. Annotations of moso bamboo genes were fed into the KEGG Pathway Tools, which is an alternative approach to categorize genes functions with an emphasis on biochemical pathways [93].

### Quantitative real-time PCR (qRT-PCR)

To evaluate the validity of Illumina analysis and assess the expression profiles in terms of specific mRNA abundances, 12 putative genes were selected and detected by qRT-PCR, and to further investigate the expression profiles of these genes, qRT-PCR of different heights shoots, was performed separately using culms after leaf expansion as the control. Reverse transcription reactions were performed using 2 mg of RNA by M-MLVRT (Promega, USA) according to the manufacturer's instructions. Sequences of 12 selected genes were obtained from the moso bamboo genome database (http://www.ncgr.ac.cn/bamboo). Primers, picked by using the Primer 3 software (http://www.genome.wi.mit.edu/cgi-bin/primer/primer3.cgi), as shown in Table S3. Tonoplast intrinsic protein (*TIP41*), cited from Fan et al. [94], were used as the internal housekeeping gene control. Real-time PCR reactions were carried out with LightCycler480® System (Roche, USA) using SYBR® Premix EX Taq™ kit (Roche, USA).The 20 µL reaction mixture contained 0.4 µL (10 µM) of each primer and 2 µL (50 ng) cDNA and 10 µL SYBR Green I Master according to the manufacturer's instructions. Amplification reactions were performed as the following: 95°C for 10 s, 60°C for 10 s, and 72°C for 20 s. All reactions were performed in triplicate, both technical and biological. Data was analyzed using Roche manager software.

### Accession code

The Illumina HiSeq™ 2000 sequencing data for moso bamboo transeriptome have been deposited in NIH Short Read Archive database under the accession number SRP029431.

## Acknowledgments

We thank Mr. Yanjun Ma, Mr. Wei Ge and Mr. Zhongyuan Sun for various supports, we also thank the sampling assistance provided by Prof. Zhongneng Wu and Mr. Junlong Liu in harvesting the materials, and we thank Ms. Anna McGurk and Dr. Jinhe Fu for their language polishment.

## Supporting Information

Figure S1 **Randomness of reads mapped to reference genome and genes.**
(TIF)

Table S1 **GO classification of differentially expressed genes.** A list of the plant-specific GO terms enriched for differentially expressed genes showing higher transcript abundance in moso bamboo shoots.
(XLS)

Table S2 **KEGG pathway annotation of differentially expressed genes.** A list of KEGG pathways enriched for differentially expressed genes showing higher transcript abundance in moso bamboo shoots.
(XLS)

Table S3 **Selected genes and primers used in qRT-PCR analysis.**
(DOC)

Table S4 **Putative genes in moso bamboo.** A list of genes putatively related to fast growth of moso bamboo shoots and their possible functions.
(XLS)
