# Supplementary figures and images for: Transcriptome Sequencing and Analysis of the Fast Growing Shoots of Moso Bamboo (Phyllostachys edulis)
Source: PLoS One. 2013 Nov 7;8(11):e78944. doi: 10.1371/journal.pone.0078944 (PMC3820679; doi:10.1371/journal.pone.0078944)

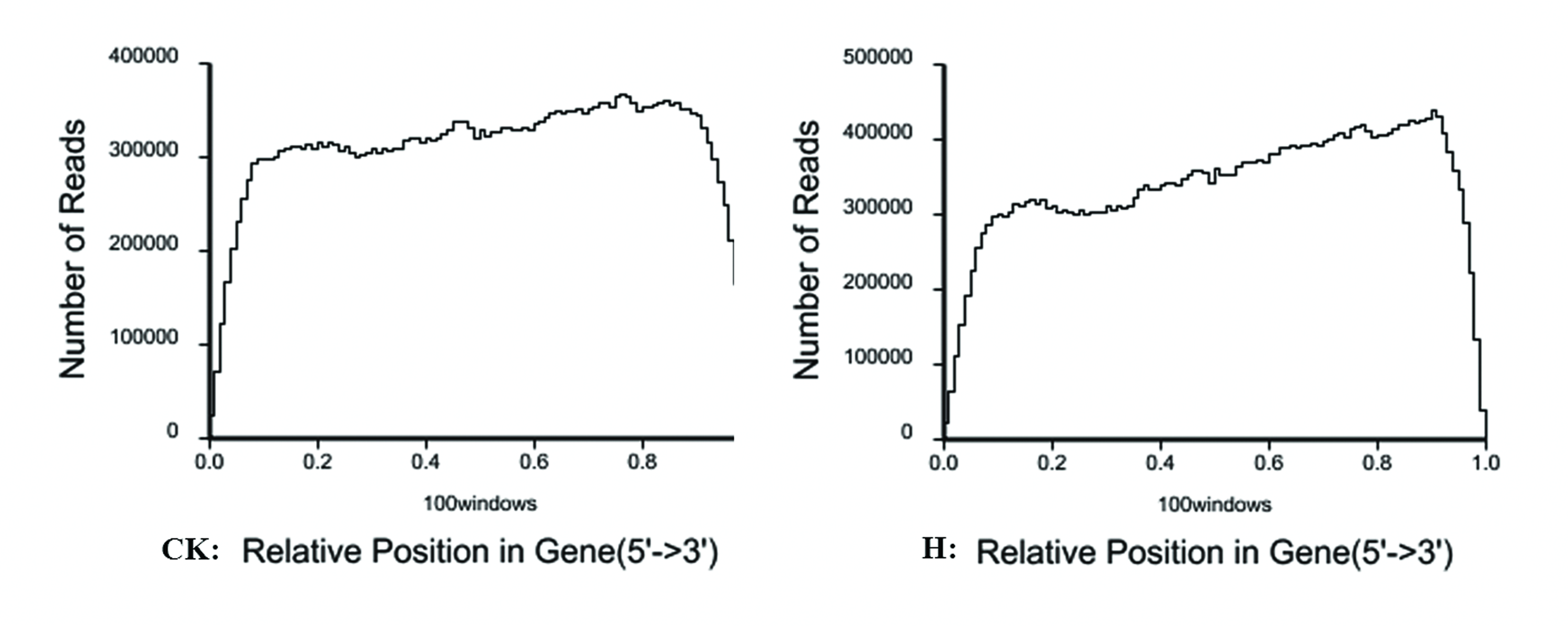

Supplement: Figure S1 — Randomness of reads mapped to reference genome and genes. (TIF) [file pone.0078944.s001.tif]
