# Supplementary material for: Transcriptome Sequencing and Analysis of the Fast Growing Shoots of Moso Bamboo (Phyllostachys edulis)
Source: PLoS One. 2013 Nov 7;8(11):e78944. doi: 10.1371/journal.pone.0078944 (PMC3820679; doi:10.1371/journal.pone.0078944)
Supplement: Table S3 — Selected genes and primers used in qRT-PCR analysis. (DOC) [file pone.0078944.s004.doc]

**Table S3.** Selected genes and primers used in qRT-PCR analysis.

| **Gene** | **Gene ID** | **Primers (5’-3’)** | **Amplication size (bp)** |
| --- | --- | --- | --- |
| *CYCA* | PH01002854G0180 | F : CGGCCTCAGCTATTTTCTTG | 156 |
|  |  | R : AGTGCTTCTTGGCGACAAAT |  |
| *EXP* | PH01002238G0310 | F : CATCAACGGCCACTCCTACT | 152 |
|  |  | R : CCAGGTACGAGTTGCTCTGC |  |
| *FTK* | PH01001577G0100 | F : GTCCTCCTCGTAGCGAACAC | 218 |
|  |  | R : CGCTCTTGCCACAGTAGTTG |  |
| *BGC* | PH01001888G0390 | F : AAATCCAACGGCATCAACTC | 200 |
|  |  | R : CAGACGTACCGGAAGGAGAC |  |
| *ARF* | PH01000057G1420 | F : GCAAGAAGGGCTTGTCTCAC | 213 |
|  |  | R : CATTTTGTTCGACCTGCTCA |  |
| *MYB* | PH01002707G0220 | F : GGAACTCGTGCCTCAAGAAG | 226 |
|  |  | R : ATTGTTGGGACGTTGGTCAC |  |
| *MYC* | PH01000201G0400 | F : GGTTTCTTCACCTTCCCACA | 239 |
|  |  | F : CATCAACCGTGGTCATGTTC |  |
| *Dof* | PH01000664G0640 | F : GCGCTTGAAGAGCTCAACTT | 220 |
|  |  | R : CCTCCAGTCCTTGACAGCTC |  |
| *SAUR* | PH01004171G0020 | F : GCCAACTTGAAGCAGATCCT | 230 |
|  |  | R : AAGACGGCCTCATTGCAG |  |
| *AUX1* | PH01000373G0290 | F : GTCATCCAGTGGTTCGAGGT | 237 |
|  |  | R : GGACCAGATGCGGTAGTTGT |  |
| *GID* | PH01000436G0270 | F : GGCACCGTCAACTGCTTACT | 181 |
|  |  | R : CGTGGGCGTAGACAATGAC |  |
| *GID1* | PH01000068G0110 | F : GACTTCTCACGCTGCTTCCT | 151 |
|  |  | R : GCTTTTCACCACCGAAGAAC |  |
| *TIP41*^a^ | — | F : AAAATCATTGTAGGCCATTGTCG | 102 |
|  |  | R : ACTAAATTAAGCCAGCGGGAGTG |  |

a The primers were cited from Fan *et al*. (2013).
